# Serum procalcitonin, C-reactive protein, and neutrophil gelatinase-associated lipocalin in early diagnosis of acute kidney injury after upper urinary tract calculi

**DOI:** 10.12669/pjms.39.1.6694

**Published:** 2023

**Authors:** Shirui Hao, Yin Guo, Ruiyan Zhao, Ting Wang, Shujun Li

**Affiliations:** 1 Shirui Hao, Department of Clinical Laboratory, Xingtai People’s Hospital, 16 Hongxing Street, Xingtai, 054001, Hebei Province, P.R. China; 2 Yin Guo, Department of Clinical Laboratory, Xingtai People’s Hospital, 16 Hongxing Street, Xingtai, 054001, Hebei Province, P.R. China; 3 Ruiyan Zhao, Department of Clinical Laboratory, Xingtai People’s Hospital, 16 Hongxing Street, Xingtai, 054001, Hebei Province, P.R. China; 4 Ting Wang, Department of Clinical Laboratory, Xingtai People’s Hospital, 16 Hongxing Street, Xingtai, 054001, Hebei Province, P.R. China; 5 Shujun Li, Department of Clinical Laboratory, Xingtai People’s Hospital, 16 Hongxing Street, Xingtai, 054001, Hebei Province, P.R. China

**Keywords:** Upper urinary calculi, Acute kidney injury, Procalcitonin, C-reactive protein, Neutrophil gelatinase-associated lipocalin

## Abstract

**Objective:**

To investigate the value of serum procalcitonin(PCT), C-reactive protein(CRP), and neutrophil gelatinase-associated lipocalin(NGAL) in the early diagnosis of acute kidney injury(AKI) after upper urinary tract calculi(UUTC).

**Methods:**

The clinical data of 86 patients who underwent UUTC surgery in our hospital from March 2020 to April 2021 were analyzed retrospectively(Approval number: 20211205L, Date: 2021-12-21). Patients were divided into an AKI group (AKI≥7 days after the operation) and a Non-AKI group. PCT, CRP, and NGAL concentrations were compared before and two hours after the operation. Multivariate logistic regression analysis was used to identify risk factors affecting the early occurrence of AKI post-operation. The receiver operating characteristic curve evaluated PCT, CRP, and NGAL in the early AKI diagnosis.

**Results:**

A total of 86 patients (30 with AKI and 56 with Non-AKI) were included. Kidney injury molecule-1(KIM-1) and urinary microalbumin(mAlb) concentrations were significantly higher in the AKI group (*P*<0.05). PCT, CRP, and NGAL concentrations were significantly higher two hours after the operation than before the operation (*P*<0.05). KIM-1 levels and elevated PCT, CRP and NGAL concentrations affected the establishment of AKI after UUTC. The sensitivity of PCT, CRP, and NGAL in evaluating AKI after UUTC were 81.17%, 84.42%, and 79.02%; the specificity was 62.31%, 71.48%, and 73.32%; and the AUC was 0.812, 0.885 and 0.804 respectively.

**Conclusions:**

PCT, CRP, and NGAL concentrations in patients with AKI after UUTC were significantly increased two hours after the operation, which can be used for the early diagnosis of AKI after UUTC operation.

## INTRODUCTION

Upper urinary tract calculi refer to kidney and ureteral calculi. Clinically, they are characterized by costal spine angle, lumbar and abdominal pain, hematuria, and bladder irritation.^1^ Surgery is an effective modality for the treatment of UUTC. UUTC can be complicated with acute kidney injury (AKI), clinical intervention after the early diagnosis of AKI is the key to shortening the treatment cycle, after the diagnosis is clear, the obstruction should be relieved as soon as possible, the urine should be drained, the infection should be controlled, and the renal function should be restored.^2^

In the past, blood urea nitrogen and blood creatinine were often used to diagnose AKI, but their concentration changes were related to the glomerular filtration rate and do not accurately reflect renal tubular injury and necrosis. The sensitivity and specificity of early diagnosis of AKI are low, necessitating the exploration of more effective biological indexes as the focus of current clinical research.^3^,^4^ The pathological characteristics of AKI are renal tubular epithelial injury, inflammation, and vascular dysfunction. Recently, biomarkers such as serum procalcitonin (PCT), C-reactive protein (CRP), and neutrophil gelatinase-associated lipocalin (NGAL) are established as markers of renal tubular and kidney function.^5^ Previous studies have found that PCT and NGAL are elevated much earlier than traditional renal function indexes such as blood urea nitrogen and blood creatinine, in the early stage of AKI after cardiac surgery. However, it is not clear whether this trend is observed following other operations.^6^,^7^

The purpose of this study was to explore the value of serum PCT, CRP, and NGAL in the early diagnosis of AKI after UUTC, to provide a theoretical basis for reducing the risk of AKI and improving prognosis.

## METHODS

The clinical records of 86 patients who underwent UUTC surgery in our hospital from March 2020 to April 2021 were analyzed retrospectively. They were divided into the AKI group and the non-AKI group according to whether AKI occurred within seven days after the operation.

### Inclusion criteria:

- Successful operation of UUTC;
- No malignant tumor, severe burn, or other serious diseases;
- Complete clinical data;

### Exclusion criteria:

- Hospital stay ≤ seven days;
- Abnormal liver and kidney function and use of nephrotoxic drugs during hospitalization;
- Previous medical history of bipolar disorder, anxiety disorder, or other mental diseases;

### Ethical approval:

This study was reviewed and approved by the medical ethics committee of Xingtai People’s Hospital (Approval number: 20211205L, Date: 2021-12-21).

### AKI diagnostic criteria:

According to the standard guidelines outlined by Kidney Disease; Improving Global Outcomes (KDIGO),^8^ renal function decreases rapidly within seven days after the operation, blood creatinine increases by 26.5μmol/L or more within 48 hours, blood creatinine increases to 1.5 times the basic value, or the urine volume is less than 0.5 ml / (kg·h) for 6~12 hours.

### Patient Characteristics:

The gender, age, BMI (body mass index), smoking history, admission signs [blood pressure, APACHE II score], operation time, and intraoperative bleeding volume were recorded.

### Laboratory indexes:

Kidney injury molecule-1 (KIM-1), serum calcium (SC), blood uric acid (BUA), urinary microalbumin (mAlb), and glycosylated hemoglobin (HbA1c) were recorded. Detection methods: Five ml of fasting venous blood was collected and centrifuged for 15 minutes. The serum and plasma were separated, and the serum was divided into two equal parts. The level of KIM-1 in serum was detected by enzyme-linked immunosorbent assay (mlbio, Shanghai, China), and the levels of SC, BUA, and HbA1c in serum were detected by an automatic biochemical analyzer. The kit was purchased from Shanghai enzyme-linked Biotechnology Co., Ltd. 24-hour urine samples were collected by gc-1200γ. A radioimmunoassay counter (Shanghai Wujiu automation equipment Co., Ltd.) detected the 24-hour mAlb levels.

Serum PCT, CRP, and NGAL concentrations before and two hours after operation were determined. Detection method: Ten ml of fasting venous blood was collected and centrifuged for 15 minutes (centrifugation radius of 12.5 cm and centrifugation speed of 3000 r/minute), and the serum was separated into three parts, labeled as No. 1, 2, 3, and stored at –20°C. Serum PCT concentrations (No.1) were detected by the double antibody sandwich immune chemiluminescence method (American Beckman Coulter company), serum CRP concentrations (No.2) were detected by an automatic biochemical analyzer (American Beckman Coulter company), and serum NGAL concentrations (No.3) were detected by solid-phase sandwich enzyme-linked immunosorbent assay (mlbio, Shanghai, China).

### Statistical analysis:

SPSS 26.0 statistical software was used to analyze experimental data and test standards α=0.05. The measurement data are described in (*χ̄*±*S*) and a t-test was used. The counting data were described by [n, (%)] and *χ^2^* test was used. Multivariate logistic regression analysis confirmed the risk factors of AKI after UUTC. The receiver operating characteristic curve (ROC) evaluated PCT, CRP, and NGAL in the early diagnosis of AKI after UUTC.

## RESULTS

According to the diagnostic criteria of AKI, the incidence of AKI within seven days after UUTC was 34.88% (30/86). A total of 30 cases were included in the AKI group, and the other 56 cases were included in the non-AKI group. There was no significant difference in sex ratio, age, BMI, smoking history, admission signs, operation time, intraoperative blood loss, SC, BUA, or HbA1c levels between the two groups (*P*>0.05). The concentrations of KIM-1 and mAlb in the AKI group were significantly higher than those in the non-AKI group (*P*<0.05), Table-I.

**Table-I:** Clinical data in the AKI and Non-AKI groups [(*χ̅*±*S*),*n*(%)].

Factor	AKI group (n =30)	Non-AKI group (n=56)	t/	P
Gender [Male, *n*(%)]	20 (66.67)	38 (67.86)	0.124	0.725
Age (year)	41.50±6.00	42.66±6.20	0.836	0.405
BMI (Kg/m^2^)	21.83±1.58	22.03±1.49	0.566	0.573
Systolic pressure (mmHg)	126.28±6.64	125.31±6.50	0.655	0.514
Diastolic pressure (mmHg)	84.61±2.64	84.49±2.30	0.219	0.827
APACHE II score	12.36±1.28	12.18±1.19	0.651	0.517
Operation time (minute)	42.15±5.16	41.18±5.18	0.848	0.399
Intraoperative bleeding (ml)	51.27±10.07	52.09±9.62	0.368	0.714
KIM-1 (ng/ml)	12.39±1.41	7.81±0.98	17.511	<0.001
SC (mmol/L)	2.23±0.38	2.20±0.36	0.407	0.685
BUA (μmol/L)	194.95±12.16	196.61±12.18	0.602	0.549
mALB (mg/24h)	506.28±63.15	226.18±70.03	18.277	<0.001
HbA1c (%)	10.03±1.02	9.97±1.04	0.277	0.783

PCT, CRP, and NGAL concentrations were significantly higher in the AKI group as compared to the non-AKI group (*P*<0.05), Table-II. Multivariate logistic regression analysis showed that the up-regulation of KIM-1 and the elevated concentrations of PCT, CRP, and NGAL were the associated risk factors of AKI after UUTC surgery, Table-III.

**Table-II:** PCT, CRP, and NGAL concentrations in the AKI and Non-AKI groups (*χ̅*±*S*).

		PCT (ng/mL)		CRP (mg/L)		NGAL (ng/mL)	

Group	n	Before operation	two hours after the operation	Before operation	two hours after the operation	Before operation	two hours after the operation
AKI group	30	0.49±0.09	7.04±1.61^a^	5.75±1.01	14.16±1.93^a^	41.26±3.22	109.28±7.28^a^
Non-AKI group	56	0.48±0.10	2.06±0.57^a^	5.68±0.98	10.27±1.23^a^	41.41±3.18	44.25±3.50^a^
*t*	-	0.482	20.936	0.296	11.382	0.219	55.971
*P*	-	0.631	<0.001	0.768	<0.001	0.827	<0.001

***Note:***

a compared with the same group before an operation, *P* < 0.05.

**Table-III:** Associated risk factors of AKI after UUTC surgery.

Independent variable	β	SE	Waldx^2^	P	OR	95%CI
KIM-1	0.700	0.304	9.915	<0.05	1.949	1.696~2.291
PCT	0.732	0.280	6.205	<0.05	2.135	1.980~2.267
CRP	0.741	0.343	6.896	<0.05	2.263	2.102~2.544
NGAL	0.703	0.375	5.914	<0.05	2.232	1.968~2.626

**Fig.1:**
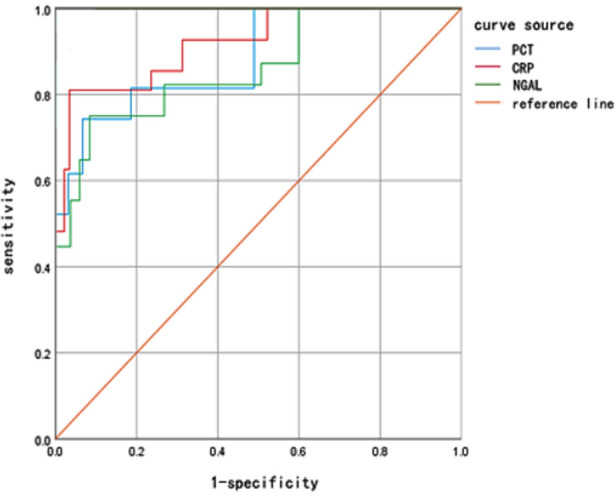
ROC curve evaluating the early diagnostic value of PCT, CRP, and NGAL in AKI after UUTC operation.

The ROC curve showed that the sensitivity of PCT, CRP, and NGAL in evaluating AKI after UUTC were 81.17%, 84.42%, and 79.02% respectively. The specificity was 62.31%, 71.48% and 73.32%, and the AUC were 0.812, 0.885 and 0.804 respectively, Fig.1.

## DISCUSSION

The postoperative situation of 86 patients with UUTC was analyzed. The incidence of AKI within seven days was about 34.88% (30/86). It shows that during the treatment of UUTC, factors such as high renal pelvic pressure, oxidative stress response and mechanical injury can cause varying degrees of damage to the renal parenchyma, resulting in the rapid loss of renal function, accompanied by pain, hematuria and other symptoms, namely AKI.^9^ Giusti G et al 10 reviewed 316 patients who underwent ureteroscopy and laser lithotripsy, and 92 (29.1%) had complications. At the same time, the review study of Cindolo I et al 11 shows that the main complication of patients with urinary calculi is renal injury. In addition, Warren J et al 12 conducted an RCT involving 1406 patients undergoing coronary artery bypass grafting, and the results showed that 449 patients (31.9%) developed AKI. It can be seen that the incidence of AKI after UUTC is similar to that of heart surgery such as valve replacement and coronary intervention.^13^ Multivariate logistic regression analysis showed that KIM-1, PCT, CRP, and NGAL were the risk factors for AKI in patients with upper urinary tract stones, suggesting that they can be used as urinary biomarkers for the diagnosis of AKI. Brewin A et al 14 showed in a review study including 12 control experiments that KIM-1, NGAL, N-acetyl-B-D-glucosamindase (NAG), protein/peptide, cytokines, and CA19-9 can be used for the diagnosis, prognosis, and stone treatment response of patients with renal injury. Serum creatinine is a marker for early clinical diagnosis of AKI but has recently been found to be affected by many factors such as age and BMI. It is particularly important to explore the biomarkers with obvious changes in the early stage of renal injury. 15

In this study, the levels of KIM-1 and mALB in the AKI group were higher than those in the non-AKI group. While KIM-1 is rarely expressed in normal renal tissue, after the injury of renal proximal convoluted tubular epithelial cells, KIM-1 is involved in the process of early renal injury and repair or the fibrosis of renal interstitium.^16^ Domestic scholar Wang JJ et al 17 found that the level of KIM-1 in AKI patients increased significantly at three, six, and 12 hours after operation in a prospective multicenter study including 149 patients. This suggests that the increased KIM-1 concentrations are related to renal injury. After AKI, the negatively charged sialic acid, heparan acetyl sulfate, and other substances in the glomerular basement membrane decrease, interfering with the affinity between proteoglycan and extracellular matrix. At this time, the filter holes on the glomerular filtration membrane increase, the negatively charged components of the glomerular filtration membrane change, and increase the discharge of mAlb.^18^

Recent studies have found that AKI after UUTC surgery is related to ischemia-reperfusion injury, oxidative stress response, blood flow mechanical trauma, inflammatory cascade, and other factors.^19^ While PCT and CRP, inflammatory response indicators, participate in oxidative stress and the inflammatory cascade and are related to apoptosis, and necrosis of renal tubular epithelium and endothelial cells.^14^ NGAL is a widely studied marker of renal tubular injury, which is mainly expressed in distal renal epithelial cells and can directly reflect the degree of renal injury.^20^ Westhoff JH *et al* 21 found that NGAL has good diagnostic performance in predicting the mortality of AKI children with different etiologies. However, Akpinar C et al^7^ found that the concentration of NGAL in blood and urine can predict the occurrence of AKI, but it is not clear whether it has the same predictive value for the early diagnosis of AKI after surgery.

The ROC curve showed that the sensitivity of PCT, CRP, and NGAL in evaluating AKI after UUTC were 81.17%, 84.42%, and 79.02% respectively. While the specificity was 62.31%, 71.48%, and 73.32% and the AUC was 0.812, 0.885, and 0.804 respectively. These results are in agreement with Leditzke K *et al* 22 suggesting that NGAL can be used as a direct and independent early biomarker of AKI after trauma in patients with severe injury. It is suggested that PCT, CRP, and NGAL concentrations can be used as biological indexes for the early diagnosis of AKI two hours after the operation of UUTC. However, due to the stepwise development of AKI, it is unclear whether PCT, CRP, and NGAL concentrations two hours after operation have the same diagnostic efficacy for middle and late AKI.^14^

### Limitations.

The sample size is small limiting the overall effect and clinical relevance. Additionally, large sample and multicenter studies are needed to explore the diagnostic efficacy of PCT, CRP, and NGAL in AKI.

## CONCLUSION

PCT, CRP, and NGAL concentrations measured two hours after the operation of upper urinary calculi can be used as biomarkers for early diagnosis of AKI. The results of this study can provide a diagnostic reference for clinicians treating AKI.

### Authors’ contributions:

**SH** conceived and designed the study.

**YG, RZ, TW and SL** collected the data and performed the analysis.

**SH** was involved in the writing of the manuscript and is responsible for the integrity of the study.

All authors have read and approved the final manuscript.
